# Investigation of Possible Sources of Electrodermal Activity in Surgical Personnel to Assess Workplace Stress Levels

**DOI:** 10.3390/s24227172

**Published:** 2024-11-08

**Authors:** Merle Schlender, Verena Uslar, Stefan Uppenkamp, Navid Tabriz, Dirk Weyhe, Timur Cetin

**Affiliations:** 1 University Medicine Oldenburg, Faculty VI, Carl von Ossietzky University Oldenburg, 26129 Oldenburg, Germany; navid.tabriz@uni-oldenburg.de (N.T.); dirk.alfons.weyhe@uni-oldenburg.de (D.W.); timur.cetin@uni-oldenburg.de (T.C.); 2 Department of Medical Physics and Acoustics, Faculty VI, Carl von Ossietzky University Oldenburg, 26129 Oldenburg, Germany; stefan.uppenkamp@uni-oldenburg.de

**Keywords:** stress level, workload, electrocardiogram, surgery

## Abstract

Objective: The aim of this study was to evaluate alternative measurement points to assess stress levels during surgery, given that taking electrodermal activity (EDA) measurements from the palms, a commonly used standard, is impractical in surgical contexts. Methods: A combination of mentally challenging tasks on a laparoscopy trainer (LapSim) and physically challenging sports exercises were performed by a group of participants. During these tasks, EDA was measured at three different locations: the fingers, toes, and shoulder/neck area. Additionally, an electrocardiogram was used as an objective measure of stress levels. Results: The findings indicated that EDA measurements taken at the toes produced similar high skin conductance levels to measurements taken at the palms. However, significantly lower skin conductance levels were observed at the shoulder. Despite this, the cross-correlation of EDA data revealed high correlation coefficients (above 0.96) between both the toe and shoulder measurements when compared to the palm data. Conclusions: The study concludes that both the toes and the shoulder/neck area serve as viable alternative locations for assessing occupational stress levels in surgical personnel. This assertion is supported by the similarity of the data produced by these methods to that of the standard finger-based technique, with the latter still being identified as the most sensitive method for capturing EDA. Subsequently, these findings attest to the potential for practical adaptation of this technique in surgical contexts.

## 1. Introduction

Surgeons are exposed to numerous different intraoperative stressors, such as time pressure or mental exertion [1]. These stressors affect surgical performance and contribute to an increase in patient complications [2].

In addition, given that high occupational stress can lead to psychological problems in surgeons [3], this highlights the importance of finding objective measures for monitoring stress levels over longer time periods in an unobtrusive way. Only when information about stress in relation to various factors is available will it be possible to develop meaningful methods for stress reduction. Subjective measures, such as the NASA (TLX) [4] or SURG (TLX) [5], have already been successfully utilized in surgery [6]. However, the problem remains that the subjectivity of the perception of stress is a limitation, but various measurement methods can be utilized to try to objectify stress. One possibility is the use of physiological measurements such as electroencephalography, skin temperature, respiration rate, electromyography, and specific skin conductance responses [7]. A classical objective measure for assessing the current stress level is the heart rate [8]. However, when measuring the heart rate, it is not possible to distinguish whether it indicates purely psychological and/or physical stress, as the body reacts with an increased heart rate in both cases [9,10]. A way to avoid physiological alterations is the use of electrodermal activity (EDA) [11].

In addition to the advantage of measuring psychologically induced stress through EDA, this method offers a simple and quick application process. In this study, EDA was chosen as the measurement method because it allows for the continuous monitoring of stress levels during long surgeries. One advantage of EDA over methods such as EEG is that it only requires the placement of electrodes, without the risk of the conductive paste drying out over extended measurement periods, as can happen with EEG. Furthermore, the application of the electrodes is much quicker and easier, meaning that it does not disrupt clinical workflows, and there is no need to factor in additional time for surgeons. Additionally, the system is small and often portable, making it easy to use during surgery without restrictions, aside from the positioning of the associated sensors.

EDA quantifies the activity of the sweat glands and the skin conductance level (SCL), measured in SI-unit Siemens. It can be used to determine psychological states, such as stress [12,13]. The EDA is typically sampled from the palmar surfaces of the hands [11,14]. These locations are recognized for displaying pronounced EDA, as discussed in previous studies by Venables and Christie [15] and Edelberg [16]. This heightened activity can be attributed to the high density of sweat glands present on the palms and soles of the feet [17,18,19], which are primarily activated by emotional arousal [11]. Multiple electrode placements can be used for EDA measurements on the palmar surfaces of the hands. One placement involves the volar surfaces of the medial phalanges, which refers to the inside of the middle finger joints. Another approach also places the electrode on the inside of the hand, specifically the distal phalanges, corresponding to the fingertips. In both methods, one electrode is positioned on the index finger, whereas the other electrode is on the middle finger. Alternate electrode placement can be made on the thenar and hypothenar eminences. In this scenario, one electrode is positioned on the fleshy mound of the palm at the base of the thumb, and the second electrode is placed on the opposite side of the wrist. Even though these placements allow for electrodermal recording, it is important to note that the recorded values may not always be consistent [14].

Skin conductance is influenced by various factors, such as the sweat output of individual glands, the signaling nerves, and, most importantly, the density of sweat glands at specific points on the skin [20,21]. Additionally, not all sweat glands in the body are regulated in the same manner. For example, beyond thermal stimuli, emotional stimuli can also activate the sympathetic nervous system, leading to signal transmission to the sweat glands [22].

However, performing measurements on the palms presents a problem during surgery, as no recordings can be performed on the hands of the surgical staff so as not to impede the surgeons in their work and, thus, not compromise patient safety.

Consequently, EDA measurement cannot be conducted during surgery, and studies investigating stress in the OR have always had to resort to subjective measurements. Alternative recording sites for EDA have been proposed in some studies, aiming to substitute finger-based measurements [20,23,24]. Edelberg [16] presented an overview of different anatomical locations for EDA recording, including positions on the fingers, hand, shoulders, neck, and chest. Another finding reported by Edelberg [16] indicates that the scalp displays higher conductance than the palmar regions of the fingers, primarily due to the high density of hair follicles. However, non-palmar areas, except for the feet, were observed to exhibit long periods of electrodermal inactivity, deeming them unsuitable for tracking EDA [25]. Furthermore, Edelberg [16] identified a promising EDA response on the medial side of the sole of the foot. In such cases, one electrode should be placed over the abductor hallucis muscle, and the other placed just below the ankle. These positions also offer the benefit of not being subjected to pressure while standing or walking [16]. Numerous other studies also explored alternative positions for deriving EDA from the soles of the feet. Payne et al. [26] compared various recording positions on the feet and deduced that the toes are remarkably comparable to the fingers in terms of EDA. Thus, as of now, the toes present the most promising alternative when hands are not available for EDA measurement.

Hossain et al. [27] demonstrated that the feet represent a viable alternative site for EDA measurement, showing a strong correlation with the standard finger-based method. Furthermore, it was observed that the forehead exhibited high robustness against motion artifacts. However, EDA data collected from the forehead displayed considerable variability in terms of correlation with data from the fingers. This variability was similarly noted for EDA measurements taken in the neck region. While some participants exhibited relatively high correlations between the forehead or neck and the fingers, others showed very low correlations. These findings suggest that the forehead and neck are less reactive compared to EDA readings measured from either the fingers or feet, making them suboptimal as alternative recording sites [27].

Several studies have demonstrated that EDA recordings can also be derived from the wrists; however, the skin conductance at the wrists is generally much lower than that of the fingers, and often no response is observed, unlike the fingers [26,28,29,30,31]. Another reason against using the wrists for electrodermal measurement is that the sweat glands in that area primarily function for thermoregulation. Boucsein [11] demonstrated this phenomenon in a study conducted in the field of electrodermal activity. This ability distinguishes them from the sweat glands located on the palms and soles, which are mainly activated by emotional arousal [11].

Studies have also been conducted on electrode placement at the shoulder [21,32] and calf [32,33]. However, these studies did not yield conclusive results that would establish these sites as viable alternatives for measuring EDA.

These studies demonstrate an interest in alternative recording sites; however, there have not been any conclusive investigations, and, as such, none of the alternative sites have been established.

Therefore, the aim of this study is to identify a method for objectively assessing stress over prolonged periods without disrupting or intruding upon the work of the subject, e.g., surgeons. Accordingly, the study plans to investigate alternative recording sites for EDA, especially recordings from the toes and the shoulder/neck region, and compare them to recordings from the widely accepted palm recording site.

The decision to use the toes as an alternative site for EDA measurement is based on the comparable density of sweat glands found on the soles of the feet and the palms of the hands, which has been well-documented in previous studies [17,18,19]. The forehead is also known to have a relatively high density of sweat glands [18,21], making it a potential alternative site for EDA measurement. However, in this study, the forehead could not be utilized due to the constraints of a surgical setting, where placing electrodes on the forehead might interfere with the medical personnel’s tasks, potentially compromising patient safety.

Since electrodes on the soles of the feet might also be perceived as intrusive, particularly during prolonged standing, another alternative recording site was considered, with a focus on minimizing interference. The shoulder/neck area was selected for this reason, despite a lack of sufficient physiological evidence. This site has been previously employed in various studies but has not been thoroughly investigated, with inconsistent results reported [17,21,27,32].

To attain a level of validity comparable to EDA measurements on the fingers, an additional objective measure in the form of pulse recording was utilized. The objective was to explore the feasibility of using the pulse, in conjunction with the alternative EDA recording sites, as a plausible approach to assessing stress levels that is equal to or is, arguably, more informative than the measurement of EDA from the palms alone. In this study, it was predicted that the measurement of EDA from the fingers would yield the most reliable results, in line with the heart rate obtained from the ECG [11]. It was also hypothesized that skin conductance values similar to those observed on the fingers would be observed owing to the similar density of sweat glands on the toes. As a result of the lower density of sweat glands near the shoulder, lower skin conductance was expected there [17]. Additionally, it was foreseen that exertion would result in an increase in both skin conductance and heart rate during EDA and ECG measurements, respectively [34]. The findings of this study could potentially be applied in future studies for the objective measurement of stress in medical staff and other occupational groups where EDA measurement at the palm is not feasible. These measurements might assist in identifying stressors, developing strategies to prevent mental health problems, and enhancing day-to-day routines in the future.

## 2. Materials and Methods

### 2.1. Participants

For this study, 20 participants (10 female and 10 male) took part in the experiment. Data from one participant was excluded from the analysis due to incomplete recording. The ages of the participants ranged from 20 to 60 years old (average age: 27 years). Participants were sourced via online notices and the total measurement period was three weeks. Initially, to assess anyone’s mental output status, all participants were required to fill out a questionnaire. This ensured or confirmed that none of the participants needed to be excluded from the study due to fatigue, stress, or lack of concentration. The study was approved by the University of Oldenburg’s local ethics committee, under local file no. 2023-127.

### 2.2. Hard- and Software

Devices from the company Plux Biosignals (explorer kit) (Plux Biosignals, Lisbon, Portugal) were utilized for the experimental setup. The 4-channel biosignalsplux hub allows the recording of raw data across 4 channels, which are transmitted to an end device via Bluetooth. This is a small, portable device that can also be used while in motion [35]. The sensors that were employed were intended for recording EDA and ECG. The biosignalsplux EDA sensor can measure the changing electrical properties of the skin. Changes in sweat secretion and sweat gland activity due to sympathetic nervous system activity can also be recorded [36]. The sensor utilized for ECG measurement possessed a low-noise local ECG differential triode configuration [37]. The sampling rate used for both EDA and ECG measurements in this study was 500 Hz.

Another technical application employed in the study was a virtual simulator (LapSim), which is a haptic-capable laparoscopy simulator from the Swedish company Surgical Sciences (Göteborg, Sweden). The validity of the LapSim device has been tested and confirmed in previous studies, including that by Woodrum et al. [38]. The Opensignals software (Version Public Build 2022-05-16, Plux, Lisbon, Portugal) was used to process the raw data. The software allows for real-time visualization of the recorded biosignals, and it is possible to record and visualize sensor data from multiple channels simultaneously [35]. MATLAB (version R2021a, Mathworks, Portola Valley, CA, USA) was used for data analysis and visualization, and IBM SPSS Statistics (version 28.0.1.0 (142), IBM, New York, NY, USA) was used for statistical analysis.

### 2.3. Experimental Procedure

Each of the participants first provided their written informed consent to participate in the study. This process included informing the participant about the experimental procedure and the management of the collected data. In addition to general information, the handedness of each participant was also determined, as this was crucial for the correct placement of the electrodes. In this study, a within-subjects design (also known as a repeated measures design) [39] was employed to compare the effectiveness of alternative methods for measuring electrodermal activity (EDA) at various body locations. In this design, measurements were taken simultaneously from the participants using both the standard method with sites on the fingers and alternative measurement sites on the feet and in the shoulder/neck region.

The choice of a within-subjects design allows for the minimization of inter-individual variability, as each participant serves as their own control.

**Figure 1:**
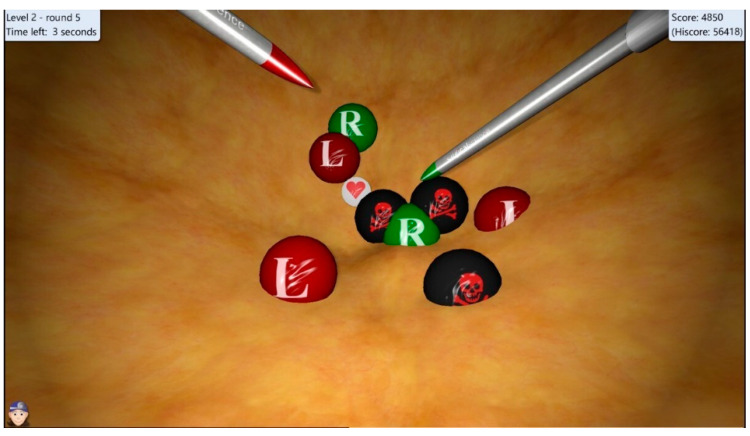
Screenshot of the LapSim task (precision and speed) for mental effort. The surgical instrument with a green tip (right) bursts the green balls (R), while the tool with a red tip (left) bursts the red balls (L). The skulls must not be hit, and the hearts bring extra points. In addition, there are blue balls, which must be touched with both tools at the same time to burst the ball.

Following the placement of the electrodes for the EDA and ECG measurements, the system was wired and established a Bluetooth connection to a laptop, then a 5-min baseline measurement was conducted, during which the participant remained static. The actual measurement encompassed two different parts, namely, mental exertion (LapSim task (precision and speed)—approximately 5 min), as shown in the screenshot in Figure 1, and physical exertion (squats—approximately 2 min). This exercise block was repeated a total of three times, with each set followed by an appropriate short break.

After the measurements were made, the electrodes and sensors were removed, and the participants completed a questionnaire, the NASA-TLX, which is reputedly used across various fields to assess subjective workload. The purpose of the NASA-TLX is to gauge the workload of employees who encounter different human–machine interface systems at work [4].

### 2.4. Statistical Analysis

Data from all the participants who completed three trials each were analyzed. Baseline-EDA data and the data corresponding to mental and physical exertion were evaluated separately. ECG data were analyzed using the Signal Processing Toolbox in MATLAB, specifically through the detection of peak values from which the heart rate was determined. To ensure accurate analysis, an individual peak detection threshold was established for each participant, excluding any peak values beneath this threshold. To avoid multiple peak detections within a single heartbeat and, thus, minimizing error, there was a requisite minimum of 60 samples between each peak detection. For EDA analysis, a three-way ANOVA with repeated measures was executed to analyze the condition (LapSim or sports tests), recording location (foot, finger, or shoulder), and trials 1–3. The acquired signals were normalized and the cross-correlations between the signals of two recording locations were calculated. To quantify the correlation coefficients from various comparisons and provide insight into the effects of each factor, another three-way repeated-measures ANOVA was carried out using the maximum value obtained from each calculated cross-correlation set, with these factors being condition, recording location, and trial. The Greenhouse–Geiser correction was used for the entire analysis because sphericity could not be assumed for all comparisons. A post hoc power analysis was conducted to assess the statistical power of the cross-correlation and ANOVA tests. This analysis was performed to evaluate the likelihood that the tests had sufficient power to detect meaningful effects, given the sample size and effect sizes observed.

## 3. Results

### 3.1. EDA Data

**Figure 2:**
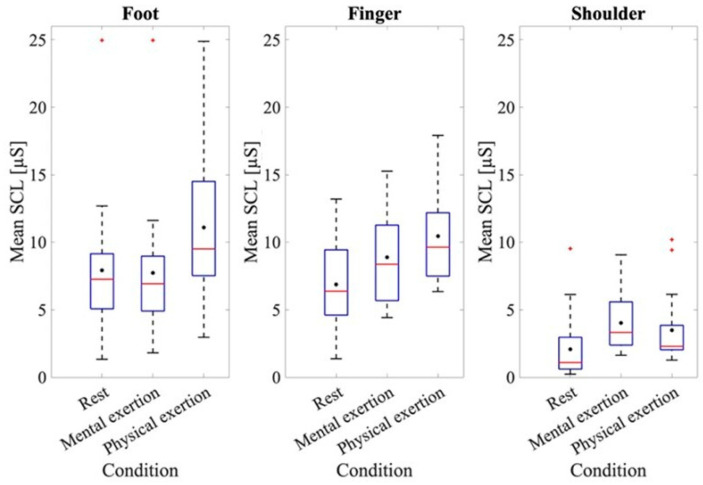
Mean SCL at rest and during physical and mental exertion for the different recording sites. The box plots show the 25th and 75th percentiles and the median in red, with the mean indicated in black.

To further examine the results of the EDA data, the results of the baseline measurement and those for each mental and physical exertion task were assessed (Figure 2). At rest, the mean SCL value for the measurement taken at the foot was 7.92 ± 5.08 μS; the measurement at the fingers was 6.87 ± 3.31 μS, while at the shoulder, it was 2.07 ± 2.38 μS.

The ANOVA of the mean baseline SCL at rest at the different recording sites indicated a significant effect for the recording site, where *F*(1.611, 28.991) = 15.899, *p* < 0.001, and partial *η*^2^ = 0.469. A significantly higher baseline SCL was found at the fingers (*p* = 0.001) and feet (*p* < 0.001) compared to the shoulder. To analyze the SCL values at the different recording sites, an ANOVA was conducted using the average skin conductance values from the different recording locations across all three trials, both during LapSim and during physical exertion. Figure 2 shows that the mean SCLs of the three recording sites were higher during physical exertion compared to those for mental exertion. Thus, the mean SCL taken from the foot during mental exertion was 7.72 ± 4.84 μS, while during physical exertion it was 11.09 ± 4.99 μS. A difference in the SCLs during mental and physical exertion can also be observed when analyzing the data from the fingers. The mean SCL during mental exertion was 8.89 ± 3.22 μS, while during physical exertion it was 10.45 ± 3.45 μS. The ANOVA for comparing the SCLs during the mental and physical exertion tests also showed a significant effect, where *F*(1, 18) = 79.88, *p* < 0.001, and partial *η*^2^ = 0.816. When examining both physical and mental exertion, it is apparent that the SCLs at the foot and finger recording sites are higher than at the shoulder recording site. The ANOVA also validated an effect for the recording site, where *F*(1.74, 31.34) = 17.53, *p* < 0.001, and partial *η*^2^ = 0.493. A subsequent evaluation using post hoc tests also showed that the shoulder recording site had significantly lower SCL values than the other two recording sites (foot: *p* = 0.001, finger: *p* < 0.001). The ANOVA also showed an effect when examining the individual trials of mental and physical exertion, where *F*(1.26, 22.67) = 9.00, *p* = 0.004, and partial *η*^2^ = 0.33. To provide a more comprehensive analysis, the interaction between the trials and recording sites was examined in more detail. The ANOVA also showed an effect here, where *F*(2.35, 42.39) = 3.99, *p* = 0.021, and partial *η*^2^ = 0.181. The post hoc tests revealed that significant differences were seen only at the shoulder recording location across all three trials (trial 1–2: *p* < 0.001, trial 2–3: *p* = 0.002, and trial 1–3: *p* < 0.001).

### 3.2. Correlation Coefficients

**Figure 3:**
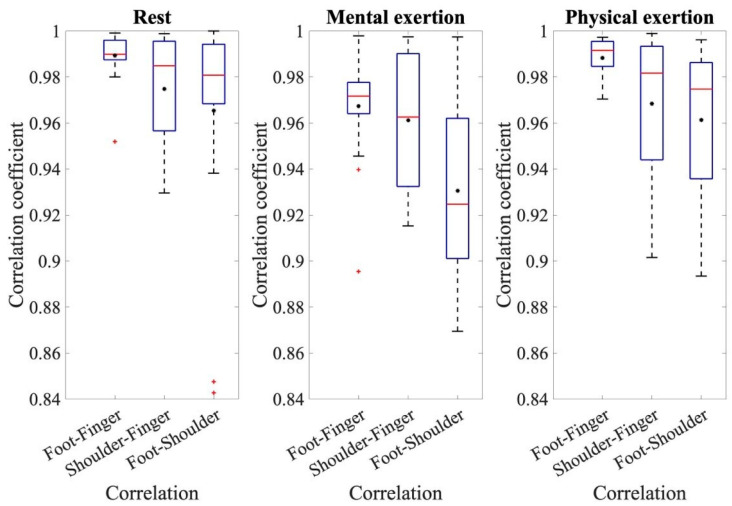
The coefficients of the cross-correlation for the resting measurement, mental exertion, and physical exertion for the comparisons of foot–finger, shoulder–finger, and foot–shoulder.

To establish a relationship between the electrodermal activity of each electrode site, cross-correlation was performed. The determined mean values and standard deviations of the correlation coefficients for the different comparisons and runs can be seen in Figure 3 as an overview.

To make assertions about the correlation coefficients, the respective correlation coefficients of the three comparisons: foot–finger, shoulder–finger, and shoulder–foot, are compared with each other for the three individual conditions: rest, mental effort, and physical effort. Figure 3 displays the coefficients of cross-correlation for the different measurements and comparative analyses. When analyzing the EDA measurements from the foot and fingers, the mean correlation coefficients are noteworthy, all being above 0.98. The correlation coefficients from the comparison of foot and finger measurements are higher than the other two comparisons and have less variation around the mean value. An ANOVA for the resting measurements demonstrated the significant effect of the correlation of the different electrode sites during rest, where *F*(1.517, 28.03) = 5.330, *p* = 0.0017, and partial *η*^2^ = 0.228. The pairwise comparisons show that the correlation coefficients of foot–finger are significantly higher than those of shoulder–finger (*p* = 0.031) and foot–shoulder (*p* = 0.044). During the analysis of the main measurement ANOVA, a significant effect from the conditions and individual correlations was also detected. The conditions referred to the different types of exertion, namely, mental and physical exertion. The correlation coefficients during physical exertion were generally higher than during mental exertion. The average correlation coefficient during mental exertion was 0.95 ± 0.047; however, during physical exertion, it was 0.97 ± 0.041. The repeated-measures ANOVA also confirmed the effect of a condition, in this case, the type of exertion, where *F*(1, 18) = 7.361, *p* = 0.014, and partial *η*^2^ = 0.290. This ANOVA also shows that the correlation coefficients were higher during physical exertion than during mental exertion. In addition, the ANOVA shows that there was an effect from individual comparisons, where *F*(1.456, 26.199) = 3.39, *p* = 0.063, and partial *η*^2^ = 0.158. The pairwise comparisons show that the correlation coefficients for the correlation between the foot and shoulder sites were significantly smaller than for the other comparisons, *p* < 0.001. Additionally, there is a significant difference in the correlation of foot–finger and shoulder–finger values, *p* = 0.030. This indicates that the correlation coefficients with foot–finger values are significantly higher than for the comparison with shoulder–finger values.

### 3.3. ECG Data

Table 1 presents the means and standard deviations of the heart rates during the different types of exertion and the resting period.

The average heart rate during physical activity was, as expected, always higher than during mental exertion. It should also be noted that the heart rate increased during both mental and physical exertion compared to the resting ECG measurement. To investigate this finding statistically, an ANOVA was conducted to determine the stages of exertion and how the differing exertion types would impact the rate of heartbeats per minute. The ANOVA showed that there is no effect of the stages on the rate of heartbeats per minute recorded, where *F*(1.980, 35.635) = 2.850, *p* = 0.072, and partial *η*^2^ = 0.137. However, the ANOVA indicated that there is an effect of the type of exertion, where *F*(1, 18) = 42.275, *p* < 0.001, and partial *η*^2^ = 0.701. Indeed, the average heart rate during physical exertion was significantly higher than during mental exertion.

### 3.4. Comparison of ECG and EDA Data

**Figure 4:**
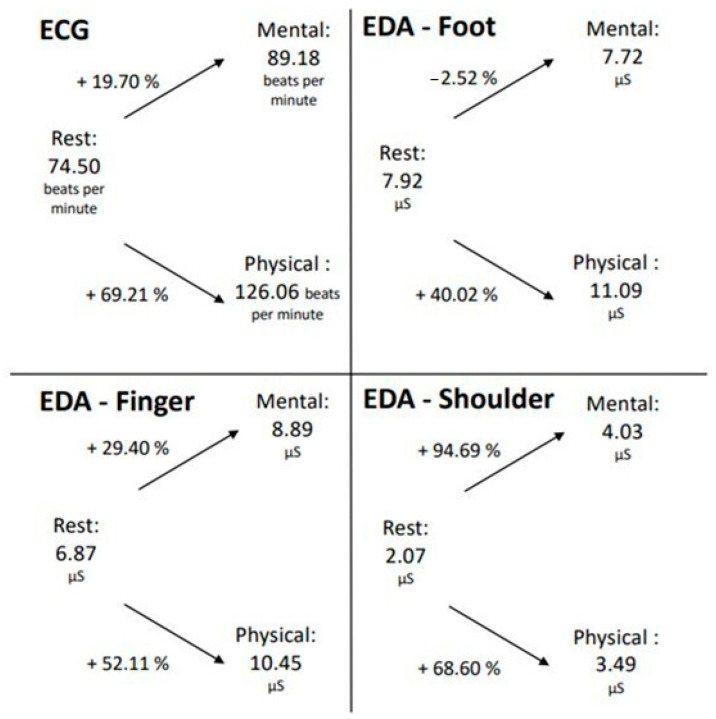
Changes in EDA and ECG data during mental and physical exertion compared to the resting measurements.

To establish a connection between the ECG and EDA data, the average EDA and ECG values, as well as the percentage change between the different conditions, were calculated. The mean value across all participants and the three trials was used for each condition of mental and physical exertion, and the mean value of all participants was used for the resting measurement (Figure 4).

In both mental and physical exertion, the number of heartbeats per minute increased relative to the resting measurement. Notably, during physical exertion, the pulse increased by 69.21%, which is significantly higher than during mental exertion (19.70%). A similar phenomenon is also observed in the EDA measurement taken from the finger. The SCL also increased during both mental and physical exertion compared to the resting measurement, with a higher increase observed during physical exertion.

However, at the foot measurement location, it is evident that the SCL decreased during mental exertion compared to the resting measurement, which contrasts with the measurement at the finger location. During physical exertion, however, there was a rise of over 50% in skin conductance registered at the foot measurement location. Therefore, for the ECG and EDA measurements from the foot and finger locations, it can be inferred that the increase in both pulse and skin conductance was greater during physical exertion than during mental exertion. The EDA measurement taken in the shoulder/neck area exhibited a different pattern. In this area, during the LapSim exercise, the SCL escalated by almost 95% during mental exertion, while only rising by 69% in the course of physical exertion. However, it should be recognized that the values of the EDA measurement in this location were generally lower than those at the foot and finger locations.

### 3.5. Post-Hoc Power Analysis

To estimate the statistical power of our data, a post hoc power analysis was conducted (G-Power, Version 3.1.9.4, Kiel University, Kiel, Germany) for an ANOVA assessing the effect of the recording site and for the analysis of correlation coefficients. For the effect of the recording site, an effect size of 0.493 was derived from the ANOVA, and the alpha level was set at 0.05 to determine the power of the statistical significance. A post hoc power analysis was also conducted for the correlation coefficients. An alpha level of 0.05 was chosen, and the effect size was set at 0.86. The rationale for this effect size was that all calculated correlation coefficients exceeded the value of 0.86.

The analysis revealed that conducting the ANOVA to determine the effect of the recording site with 19 participants achieved a power of 0.99. Similarly, a power of 0.99 was also attained for the correlation coefficients with the same 19 participants.

## 4. Discussion

The objective of this study was to ascertain if there were alternative recording sites to those used in the standardized method for measuring electrodermal activity on the finger, particularly under mental and physical stress conditions.

The study also sought to determine the feasibility of deriving pulse rate alongside EDA to assess another objective measure of stress level. Heart rate and electrodermal activity (EDA) were recorded to provide insights into the resultant stress levels and to compare different sites for EDA measurement. The EDA data were compared and correlated with heart rate for analysis.

During resting measurements, the highest level of electrodermal activity was observed at the foot, followed by the fingers, and both were significantly higher than at the shoulder, despite the absence of any exertion. Skin conductance values at the recording sites for the finger and foot were also higher than at the shoulder, corroborating the sweat gland density theory developed by Shields et al. [34].

Skin conductance values at the foot and finger were significantly higher during physical exertion than during mental exertion. Contrastingly, there was no significant variation in skin conductance values between mental and physical exertion at the shoulder. This aligns with the theory proposed by Shields et al. [34] and Sato et al. [40] that higher activity can be measured at the soles and palms due to a higher density of sweat glands, suggesting that the sweat glands at the shoulder are less sensitive or are present in lower numbers and, therefore, do not respond as robustly to stress, irrespective of the exertion level. An analysis of the cross-correlations reveals that the average correlation coefficients for the two conditions across different trials exceeded 0.9, although there were, overall, higher correlations for physical exertion than for mental exertion. This suggests that the compared signals have a high degree of similarity, thereby enabling the acquisition of comparable results from alternative electrode sites.

Given that the comparison between the foot and the finger yielded a high mean correlation coefficient and a low variation, this substantiates the theory that the foot is a reliable site for resting-state measurement, showing a strong similarity with the finger measurement. However, it must also be emphasized that the correlation coefficient between the shoulder and finger electrode sites was also significantly high. That suggests that the results obtained from the shoulder show a high degree of resemblance to those from the finger, positioning the shoulder as a potential alternative electrode site. The potential for alternative electrode sites has also been discussed by Hossain et al. [27], Kasos et al. [32], and Kasos et al. [33], although their findings did not yield conclusive results. This highlights the need for further research in this area to clarify the effectiveness of these alternative sites. The heart rate measured during physical exertion was higher than during mental exertion, suggesting that physical exertion was more strenuous than mental exertion.

Additionally, the electrocardiogram data showed that, unlike during the resting-state measurement, an increase in heartbeats per minute was observed during both forms of exertion, and there was no effect of the trials, indicating that there was a sufficient pause between the individual measurements to regulate the pulse back to resting levels.

Increases in both the heart rate and electrodermal activity suggest that the exertions performed were suitable for inducing stress, resulting in an increase in the biosignals. To compare ECG and EDA data, the percentage change between the data from the resting measurement and the various activities was analyzed. An increase in heartbeats per minute was observed during both mental and physical exertion, with the percentage change being significantly higher during physical exertion. A similar pattern was observed in the EDA measurements taken from the finger, confirming the hypothesis that physical exertion induces more “stress” than mental exertion. Increases in heart rate and finger skin conductance during both physical and mental exertion demonstrated that stress was induced during the LapSim task and knee bends. Moreover, it was shown that both ECG measurement and EDA derivation from the finger could be used to measure the stress induced by mental exertion, even if it was less than the stress induced by physical exertion. The derivation of electrodermal activity on the toe also proved effective in measuring stress.

Simultaneously, an effect was observed during physical exertion, leading to a significant increase in skin conductance and visible stress at the toe. Given the low level of stress induced during the LapSim task, it was only possible to measure it using the more sensitive measurement position on the finger, rather than the less sensitive alternative measurement position on the toe. Moreover, it seems that the derivation from the shoulder/neck area also functioned efficiently, as an increase in EDA was measured for both the LapSim task and physical activity. However, it is noteworthy that a higher increase in skin conductance was observed during mental exertion than during physical exertion, which contradicts the results of the electrodermal activity derivation from the finger and the ECG. Further research is needed to explore this discrepancy. However, deriving skin conductance at the toe and shoulder/neck area could serve as a potential alternative to the standard method of measurement at the finger. It is notable that toe-derived measurement was less sensitive than finger-derived measurement, as reflected in the heart rate comparison. Also, a comparison of the EDA and ECG data revealed that the pulse derivation showed higher sensitivity for stress measurement than the toes’ skin conductance derivation.

In conclusion, our findings support the hypothesis that EDA measurement at the finger produces the most reliable results for stress measurements over alternative locations such as the toes or shoulder/neck area.

They can also validate the theory that similar skin conductance values can be measured at the toes and fingers. This theory was confirmed by the correlation coefficients and was also evident in the measured SCL values of the different efforts, which show similarities between the foot and finger derivation sites. These results align with the study by Shields et al. [34], which showed a comparable density of sweat glands on the soles of the hands and feet, yielding similar skin conductance values.

Additionally, the results of the studies by Hossain et al. [27] and Payne et al. [41] were also confirmed, which showed that toes can represent an alternative derivation position for electrodermal activity.

However, the premise that both physical and mental effort result in an increase in EDA and ECG measurements could not be generally confirmed. Voss et al. [10] demonstrated that physical activity promotes an increase in heart rate, which was likewise observed during physical exertion in this study. Moreover, at the finger derivation site, increases in EDA data were recorded during both types of effort, which was consistent with expectations. The toe measurements, on the other hand, only detected an increase in electrodermal activity during physical exertion and not during mental exertion, despite Schumm et al. [42] indicating that stress results in increased sweat gland activity and, consequently, enhanced EDA. Therefore, it can be inferred that the toes are less sensitive to changes in skin conductance and are not as reactive to mental effort as to physical exertion.

The two electrode sites at the fingers and the feet were previously explored by Edelberg [16]. Additionally, Edelberg [16] evaluated the shoulder/neck region as an alternative recording site, but their study did not yield substantial results. Furthermore, both Payne et al. [26] and Hossain et al. [27] proposed the foot electrode site as a promising alternative.

Our study made it possible to derive electrodermal activity results from different recording sites and to compare these with the standardized method of measurement at the fingers. Hence, if the fingers are unavailable for EDA measurement, as in the case of surgical personnel, the foot and shoulder represent practical alternates.

However, in comparison to other locations, the shoulder/neck area did not produce substantial results. Consequently, the combined derivation of skin conductance from the toes and heart rate measurement appears to be an advantageous alternative when EDA recording at the finger is not viable. Another feasible option entails combining shoulder recording, which demonstrates significant similarities to finger derivation, with ECG, thus presenting the potential for obtaining trustworthy results.

There are several limitations to this study. First of all, there is the relatively small sample size of the participants. A limited number of participants was selected due to practical constraints; primarily, these were the lack of broader availability and the fact that, in future studies, fewer healthcare professionals will be available in a real operating room setting. However, the post hoc power analysis indicated that the study, with a sample size of 19, achieved a sufficient statistical power of 0.99 to address the primary research questions and to draw meaningful conclusions from the main findings. Thus, our chosen sample size was adequate for assessing the general trends and relationships, such as the correlations and group comparisons. However, for more detailed analyses, particularly those involving the complexities of ECG measurements or the interactions between different exertion levels and recording sites, a larger sample size would be required. Increasing the sample size in future studies would allow for more precise estimates and greater reliability when examining these more nuanced aspects.

Additionally, only two stressors (mental and physical) were considered in this study, as the primary focus was on a different research question. In conclusion, we successfully achieved the main objective of identifying a non-intrusive alternative site for EDA measurement that shows a strong correlation with the standard method using the fingers. The study design enabled the identification of potential alternative sites but did not focus on developing new analytical methods, which could be explored in future studies. Within this study, established methods were employed, which could be expanded to, for example, more accurately analyzing spontaneous reactions to individual signals. However, this was not the focus of this feasibility study.

In future studies, the focus will shift toward incorporating more complex, surgery-relevant stressors to further explore their impact on EDA measurements and to validate the results under a wider range of conditions. While this study primarily examined two types of stressors—mental and physical exertion—it did not address other stress conditions that are commonly encountered during surgery, such as time pressure, unexpected events, or patient complications. By providing an alternative, non-intrusive EDA measurement site, this study offers the opportunity to expand future research to more realistic surgical settings with a broader variety of stressors, facilitating validation in real-world applications. To gain a more comprehensive understanding of the effects of diverse surgical stressors, future studies should consider these additional conditions.

## 5. Conclusions

This study has demonstrated the feasibility of measuring electrodermal activity (EDA), not only through the conventional method of taking recordings from the fingers but also taking them from the shoulder and toes during surgical procedures. Furthermore, these alternative methods for EDA measurement could be applicable in various professional fields beyond surgery, such as the aerospace sector, to assess and potentially mitigate workplace stressors. To confirm the effectiveness of these alternative sites and refine the methodology, additional research should be conducted in surgical settings. As interactions between humans and technology continue to grow, particularly in surgical environments, the ability to objectively assess stress levels becomes increasingly crucial. Such objective assessments will provide valuable insights into the impact of human–technology interactions on stress, enhancing both safety and performance in high-stakes situations.

## Figures and Tables

**Table 1:** Average heartbeats per minute across all subjects during different exertion and rest periods.

Trial	Heartbeats Per Minute
Rest	74.50 ± 9.58
Mental exertion 1	87.09 ± 11.85
Physical exertion 1	124.95 ± 20.27
Mental exertion 2	88.44 ± 15.33
Physical exertion 2	126.06 ± 20.87
Mental exertion 3	92.01 ± 13.87
Physical exertion 3	127.16 ± 18.67

## Data Availability

The data presented in this study are available on request from the corresponding author.
